# Campbell title registrations to date – August 2024, and discontinued protocols

**DOI:** 10.1002/cl2.1439

**Published:** 2024-09-11

Details of new titles for systematic reviews or evidence and gap maps that have been accepted by the Editor of a Campbell Coordinating Group are published in each issue of the journal. If you would like to receive a copy of the approved title registration form, please *send an email* to the Managing Editor of the relevant Coordinating Group.

A list of discontinued protocols appears below these new titles. If you are interested to continue a project, please get in touch with the Managing Editor of the relevant Coordinating Group or email info@campbellcollaboration.org.

## AGEING

Intergenerational programs to improve the psychosocial health and wellbeing of older adults in residential aged care: What works, for whom, in what circumstances and how? A rapid realist review

Lysha Lee, Pat Nicholson, Kayla Lock, Katrin Gerber, Alison Hutchinson

23 July 2024

## CHILDREN AND YOUNG PERSONS WELLBEING

Availability and effectiveness of interventions for the prevention and treatment of obesity in children and adolescents from low‐ and middle‐income countries: Protocol for a systematic review and meta‐analysis

Gerardo Gomez, Muhammad Asim, Aliya Ayub, Zulfiqar Bhutta, Saima Afaq

3 August 2024

Effectiveness of school‐based nutrition education interventions on obesity and nutritional status of adolescent girls: A systematic review

Sehar Iqbal, Taima Qudah, Abdul Momin Rizwan Ahmad

3 August 2024

Influencing factors and strategies to improve emotional regulation among adolescents: A mixed‐method systematic review

Preejana Sharma, Shweta Rai, Linu George

24 June 2024

Interventions and outcomes for depressive symptoms among children and adolescents: An evidence and gap map.

Pengpeng Cao, Yuhao Li, Luyao Yang, Shuo Zhang, Jieyixi Hua, Bingxia Dang, Chuying Li, Bei An, Zheng Xu

24 June 2024

What does the available research tell us about health visitor activities in the United Kingdom and the outcomes for babies and children under five years of age, and their parents or primary caregivers: An evidence and gap map

Michael Fanner, Jane Barlow, Sarah Cowley, Karen Whittaker, Helen Critcher, Mary Malone

24 June 2024

Effects of positive psychological interventions on young children's mental health and well‐being: A systematic review

Daniel Hernández‐Torrano, Dianne Vella‐Brodrick, Laura Ibrayeva, Manat Sergazina, Kelsey Lewis, Anara Burambayeva, Aiida Kulsary

19 June 2024

Bereavement interventions for children and adolescents: An evidence and gap map of primary studies and systematic reviews

Leonor Rodriguez, James Lyons, Amy Maloy, Ailsa O'Brien

10 June 2024

## CLIMATE SOLUTIONS

Suicidal behaviours post‐natural disasters: An evidence gap map

Mike Trott, Urska Arnautovska, Dan Siskind

1 June 2024

## CRIME AND JUSTICE

Situational crime prevention measures to prevent terrorist attacks against soft targets and crowded places: An evidence and gap map

Caitlin Clemmow, Zoe Marchment, Paul Gill

22 August 2024

Deterrence‐oriented counterterrorism interventions in OECD countries for reducing terrorism: A systematic review

Esther Salama, Michael Wolfowicz, Badi Hasisi

8 August 2024

Effectiveness and implementation of community‐based initiatives and educational programmes to counter online violent extremist propaganda in English, French, Spanish, Portuguese, German and Scandinavian languages: A systematic review

Felipe Pathe Duarte, Cátia Moreira de Carvalho, Pedro Barbosa, Matteo Vergani

18 July 2024

Prevalence and risk and protective factors for radicalization among school‐aged youth: A systematic review

Anthony Petrosino, Claire Morgan

18 July 2024

Interventions to prevent cognitive and behavioural radicalisation: A systematic review

Sara Valdebenito Munoz, Manuel Eisner

18 July 2024

Interventions targeting misinformation, disinformation and malinformation for reducing and countering violent extremism: A systematic review

Catia de Carvalho, João Pedro Ramos, Pedro Barbosa, Sarah Carthy, Marta Pinto

9 June 2024

Non‐criminal justice interventions for countering cognitive and behavioural radicalisation amongst children and adolescents: A systematic review of effectiveness and implementation

Sarah Marsden, James Lewis, Anna Stefaniak, James Hewitt

31 May 2024

## KNOWLEDGE TRANSLATION AND IMPLEMENTATION

Comparative effectiveness of tools for communicating health research results to study participants and others with relevant lived experience: A living systematic review of randomized controlled trials

Elsa‐Lynn Nassar, Claire Adams, Danielle Rice, Amanda Wurz, Annabelle South, Jill Boruff, Marie‐Eve Carrier, Susan Bartlett, Andrea Benedetti, Katie Gillies, Agnes Kocher, Linda Kwakkenbos, Mwidimi Ndosi, Matthew Sydes, Amy Gietzen, Karen Gottesman, Geneviève Guillot, Amanda Lawrie‐Jones, Catarina Leite, John Michalski, Michelle Richard, Maureen Sauve, Brett D. Thombs

11 July 2024

## SOCIAL WELFARE

Financial coaching for improving finances and well‐being for households: A systematic review

Julie Birkenmaier, Brandy Maynard

22 August 2024

Investigating the intersection of parenting and substance use recovery identities: A scoping review

Jenny O'Connor, Shane George, Morgan Klein, Nicklaus Herbst, Melis Lydston, John Kelly, Emily Hennessy

24 July 2024

Mutual help organizations to support recovery among individuals with drug use disorders: A systematic review

Emily Hennessy, Brandon Bergman, John Kelly

13 June 2024

## DISCONTINUED PROTOCOLS

If you are interested to continue one of the projects below, please get in touch with the Managing Editor of the relevant Coordinating Group or email info@campbellcollaboration.org.

## CRIME AND JUSTICE

https://onlinelibrary.wiley.com/doi/10.1002/CL2.85

PROTOCOL: Mandatory arrest for misdemeanor domestic violence effects on repeat offending

Barak Ariel, Lawrence W. Sherman

## EDUCATION

https://onlinelibrary.wiley.com/doi/10.1002/CL2.163

PROTOCOL: Provision of information and communications technology (ICT) for improving academic achievement and school engagement in students aged 4–18

Kristin Liabo, Laurenz Langer, Antonia Simon, Kathy‐Ann Daniel‐Gittens, Alex Elwick, Janice Tripney

https://onlinelibrary.wiley.com/doi/10.1002/CL2.210

PROTOCOL: Universal school‐based programmes for improving social and emotional outcomes in children aged 3–11 years

Paul Connolly, Sarah Miller, Jennifer Hanratty, Jennifer Roberts, Seaneen Sloan

https://onlinelibrary.wiley.com/doi/10.1002/CL2.110

PROTOCOL: Education support services for improving school engagement and academic performance of children and adolescents with a chronic health condition

Michelle A. Tollit, Susan M. Sawyer, Savithiri Ratnapalan, Tony Barnett

https://onlinelibrary.wiley.com/doi/10.1002/CL2.197

PROTOCOL: Electronic mentoring to promote positive youth outcomes for young people under 25: A systematic review

Robyn M. O'Connor, David L. DuBois, Lucy Bowes

https://onlinelibrary.wiley.com/doi/10.1002/CL2.166

PROTOCOL: Merit pay programs for improving teacher retention, teacher satisfaction, and student achievement in primary and secondary education: A systematic review

Gary Ritter, Julie Trivitt, Leesa Foreman, Corey DeAngelis, George Denny

## INTERNATIONAL DEVELOPMENT

https://onlinelibrary.wiley.com/doi/10.1002/cl2.1122

PROTOCOL: Development evaluations in India 2000–2018: A country impact evaluation map

Ashrita Saran, Eti Rajwar, Bhumika T. V., Divya S. Patil, Howard White

https://onlinelibrary.wiley.com/doi/10.1002/cl2.1186

PROTOCOL: The association between whole‐grain dietary intake and noncommunicable diseases: A systematic review and meta‐analysis

Wasim A. Iqbal, Gavin B. Stewart, Abigail Smith, Linda Errington, Chris J. Seal

https://onlinelibrary.wiley.com/doi/10.1002/CL2.84

PROTOCOL: Nutrition interventions and programs for reducing mortality and morbidity in pregnant and lactating women and women of reproductive age: A systematic review

Philippa Middleton, Caroline Crowther, Tanya Bubner, Vicki Flenady, Zulfiqar Bhutta, Tran son Trach, Zohra Lassi

https://onlinelibrary.wiley.com/doi/10.1002/CL2.138

PROTOCOL: Improving maternal, newborn and women's reproductive health in crisis settings

Primus Che Chi, Henrik Urdal, Odidika U. J. Umeora, Johanne Sundby, Paul Spiegel, Declan Devane

https://onlinelibrary.wiley.com/doi/10.1002/CL2.183

PROTOCOL: The impacts of interventions for female economic empowerment at the community level on human development

Marcela Ibanez, Sarah Khan, Anna Minasyan, Soham Sahoo, Pooja Balasubramanian

https://onlinelibrary.wiley.com/doi/10.1002/CL2.154

PROTOCOL: Peer‐based interventions for reducing morbidity and mortality in HIV‐positive women

J. Petkovic, M. Doull, A. M. O'Connor, J. Aweya, M. Yoganathan, V. Welch, G. A. Wells, P. Tugwell

https://onlinelibrary.wiley.com/doi/10.1002/CL2.188

PROTOCOL: Community‐led total sanitation in rural areas of low‐ and middle‐income countries: A systematic review of evidence on effects and influencing factors

Josef Novotný, Jiří Hasman, Martin Lepič, Vít Bořil

https://onlinelibrary.wiley.com/doi/10.1002/CL2.140

PROTOCOL: Access to electricity for improving health, education and welfare in low‐ and middle‐income countries

Kavita Mathur, Sandy Oliver, Janice Tripney

https://onlinelibrary.wiley.com/doi/10.1002/CL2.157

PROTOCOL: Saving promotion interventions for improving saving behaviour and reducing poverty in low‐ and middle‐income countries

Janina Isabel Steinert, Ani Movsisyan, Juliane Zenker, Ute Filipiak, Yulia Shenderovich

https://onlinelibrary.wiley.com/doi/10.1002/CL2.172

PROTOCOL: The effect of interventions for women's empowerment on children's health and education

Sebastian Vollmer, Sarah Khan, Le Thi Ngoc Tu, Atika Pasha, Soham Sahoo

https://onlinelibrary.wiley.com/doi/10.1002/CL2.200

PROTOCOL: The effects of road infrastructure, and transport and logistics services interventions on women's participation in informal and formal labour markets in low‐ and middle‐income countries

Manisha Gupta, Souvik Bandyopadhyay, Meerambika Mahapatro, Shreya Jha

https://onlinelibrary.wiley.com/doi/10.1002/cl2.1135

PROTOCOL: Water, sanitation and hygiene for reducing childhood mortality in low‐ and middle‐income countries

Hugh Sharma Waddington, Sandy Cairncross

## SOCIAL WELFARE

https://onlinelibrary.wiley.com/doi/10.1002/cl2.1259

PROTOCOL: Effects of virtual reality exposure therapy versus in vivo exposure in treating social anxiety disorder in adults: A systematic review and meta‐analysis

Martin Polak, Norbert Tanzer, Per Carlbring

https://onlinelibrary.wiley.com/doi/10.1002/CL2.109

PROTOCOL: The therapeutic alliance and psychotherapy outcomes for young adults aged 18 to 34

Stacy J. Green, Julia H. Littell, Karianne Hammerstrøm, Emily Tanner‐Smith, Jessica Schaffner Wilen

https://onlinelibrary.wiley.com/doi/10.1002/CL2.56

PROTOCOL: Cognitive‐behavioural therapy for parents who have physically abused their children

Mogens N. Christoffersen, Jacqueline Corcoran, Diane DePanfilis, Claire Daining

